# Vehicle Load Information Acquisition Using Roadside Micro-Electromechanical Systems Accelerometers

**DOI:** 10.3390/s25164901

**Published:** 2025-08-08

**Authors:** Qian Zhao, Zhoujing Ye, Zhao Tan, Jie Xu, Linbing Wang

**Affiliations:** 1 National Engineering Research Center for Digital Construction and Evaluation of Urban Rail Transit, China Railway Design Corporation, Co., Ltd., Tianjin 300308, China; zhaoqian928@126.com (Q.Z.); tanzhao@crdc.com (Z.T.); 2 National Center for Material Service Safety, University of Science and Technology Beijing, Beijing 100083, China; yezhoujing@ustb.edu.cn; 3 School of Civil Engineering, Tianjin University, Tianjin 300350, China; 4 Key Laboratory of Coast Civil Structure Safety of the Ministry of Education, Tianjin University, Tianjin 300350, China; 5 The Sensing and Perception Lab, University of Georgia, Athens, GA 30602, USA

**Keywords:** pavement vibration, vehicle speed, vehicle load estimation, lateral position, MEMS accelerometer, Accelerated Pavement Testing (APT)

## Abstract

Vehicle load is crucial for road design, maintenance, and expansion, while vehicle speed and lateral position are essential for traffic management and driving safety. This paper introduces a method for collecting vehicle speed, lateral position, and load information using roadside Micro-Electromechanical Systems (MEMS) accelerometers located on the pavement. Firstly, this research analyzes the distribution of pavement vibration responses in both lateral and vertical directions based on the Finite Element Method (FEM) data provided in the literature. Then, pavement vibration data is collected by roadside sensors with a Full-scale Accelerated Loading Tester, considering varying vehicle speeds, loads, and lateral positions. The results reveal that the vertical peak acceleration increases linearly with vehicle speed within a range of 5–22 km/h, decreases following a power law as the lateral distance between the wheel center and sensor increases from 0.4 to 0.9 m, which is consistent with the trends observed in the literature’s FEM data. The vibration energy of the vertical acceleration exhibits a positive linear correlation with the total vehicle load, with a correlation coefficient of 0.885. This approach offers a practical method for vehicle load estimation, optimal sensor deployment, and enhancement of pavement performance monitoring systems.

## 1. Introduction

Vehicle load information is a key factor for evaluating roadway infrastructure performance and enhancing traffic efficiency. Access to detailed vehicle data—such as load, type, speed, and volume—improves road design reliability, helps optimize maintenance and rehabilitation investments, and extends service life [1]. In the era of connected autonomous vehicles, techniques like satellite navigation [2], computer vision, and Vehicle-to-Infrastructure (V2I) systems offer increasingly precise positioning capabilities [3].

From the perspective of road infrastructure, vehicle load directly impacts the design parameters of the structure. Long-term load data also reveal how repetitive stresses affect pavement performance over time [4], aiding in maintenance scheduling and preventative measures [5]. Furthermore, for road expansion and renovation plans, vehicle load information provides a scientific basis for decision-making [6]. Meanwhile, Intelligent Transportation Systems (ITS) rely on rapid, accurate vehicle information to bolster safety, reduce congestion, cut emissions, and improve passenger comfort [7].

Current sensing and perception technologies include roadside devices (e.g., vision-based methods and radar) and embedded systems such as commercial Weigh-In-Motion (WIM) sensors or strain gauges [8]. Llorca et al. [9] surveyed vision-based speed estimation methods and provided performance metrics. Zhang et al. [10] integrated cameras, thermal cameras, and radars using an edge-cloud framework to gather traffic data and enhance safety. Zhang et al. [11] obtained vehicle axle spacing and the number of axles by five stress–strain sensors embedded below the surface of a concrete pavement slab symmetrically to measure all the vehicles traveling on the road. Xue et al. [12] measured the pavement dynamic response using a group of strain gauges and pressure cells installed at the bottom of the pavement surface layer, and back-calculated the vehicle weight, speed, type by a Gaussian model using Finite Element (FE) simulation.

To address cost, longevity, and data quality issues, researchers are exploring fiber optic sensing [13,14], MEMS accelerometers, and machine-learning approaches [15,16] for vehicle load monitoring. Ye et al. [17] designed accelerometer nodes to measure pavement vibration for speed and classification. Liu et al. [18] embedded vibration accelerometers in the pavement for vehicle classification using a convolutional neural network. Chang et al. [19] embedded micro-electromechanical systems (MEMS) accelerometer sensors in PCC pavement to capture vibration signals induced by vehicles.

In addition to vertical load distribution, lateral load placement on the pavement can accelerate localized wear and deformation. Shen et al. [20] examined six levels of the vehicle, wandering from 0 cm to 30.5 cm by FEMs, which shows that the wave peak value of the signal varies from 150 to 360 kPa. Moreover, a vehicle’s lateral position is crucial for road safety, as demonstrated in several studies. Verster and Roth [21] found that the standard deviation of lateral position better reflects safe driving behavior than that of speed, and Charly and Mathew [22] showed that precise lateral positioning can help predict crashes. Zheng and Li [23] proposed a dual-layer deep neural network to estimate lateral position by computing a vehicle’s projected area.

While accurate lateral positioning remains vital for safe driving, load information is typically gathered through pavement-based WIM systems or bridge-based BWIM systems [24,25,26]. These installations are expensive and often limited to high-risk or critical locations. Meanwhile, traffic flow is usually captured via roadside equipment like cameras or radar, which can be affected by adverse weather and require substantial processing resources. In addition, load data and traffic data frequently reside in separate platforms, making integrated analysis challenging [14,27].

However, current methods for acquiring vehicle load data typically require sensors to be embedded within the pavement structure. When MEMS-based vibration sensors are used for pavement load or traffic information collection, they are usually deployed in lateral arrays to ensure the capture of vibration signals directly beneath the wheel load. Existing pavement responses to vehicle loads are primarily derived from measured stress–strain responses or simulation results, but these often fail to provide generalized pavement vibration response patterns due to differences in pavement structure and materials or the simplifications employed in simulations.

To address this, the present study first derives the impact of vehicle load and speed on dynamic vehicle loads and pavement responses using a simplified vehicle–pavement interaction model. Based on finite element simulation data from the literature, it then proposes distribution patterns of pavement vibration responses along both lateral and vertical directions. Subsequently, MEMS accelerometers are installed on the road surface along the driving lane side for pavement vibration data acquisition at various speeds, loads, and lateral positions using a full-scale Accelerated Loading Tester The method’s feasibility is demonstrated through vertical vibration signal peaks and vibration energy. Additionally, the experimental data further supports the attenuation pattern of vertical pavement vibration signals with lateral distance, as derived from the finite element simulation data in the literature. This study highlights the scientific rigor of the proposed method, the reliability of its conclusions, and its promising potential for practical application.

## 2. Methodology

### 2.1. Theoretical Analysis

#### 2.1.1. Pavement Vibration Analysis Based on Vehicle–Road Interactions

**Figure 1:**
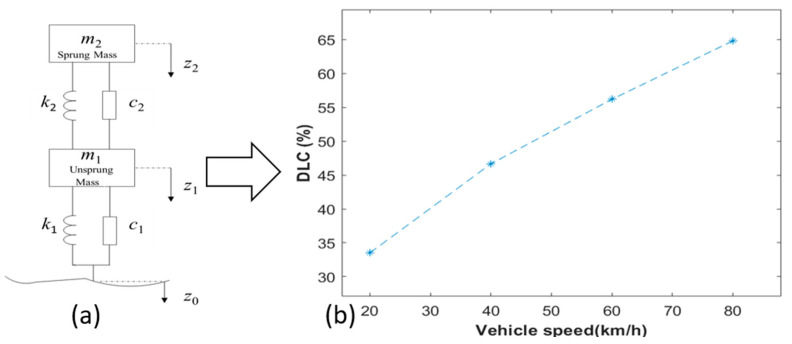
(**a**) The 2-DOF vehicle model [5]. (**b**) Vehicle–pavement DLC under different speeds.

The interaction between vehicles and pavement surfaces plays a critical role in dynamic load generation and road surface responses. Road irregularities introduce additional dynamic forces, which can be reduced through phase-optimized suspension systems enhancing road-friendliness [28]. In this study, road roughness is represented as a Gaussian random process using filtered white noise method (FWNM), which satisfies the power spectral density (PSD) defined by [29]. Compared to harmonic superposition methods, FWNM provides better computational stability and agreement with measured road spectra [30]. Subsequently, a two-degree-of-freedom (2-DOF) model was implemented (Figure 1a). Following D’ Alembert’s principle, the motion differential equation for this vehicle model can be articulated as follows:

(1) $$\left[\begin{array}{cc} m_{2} & 0\\ 0 & m_{1} \end{array}\right]\left[\begin{array}{c} \ddot{z_{2}}\\ \begin{array}{c} \ddot{z_{1}} \end{array} \end{array}\right]+\left[\begin{array}{cc} c_{2} & -c_{2}\\ {-c}_{2} & c_{1}+c_{2} \end{array}\right]\left[\begin{array}{c} \dot{z_{2}}\\ \dot{z_{1}} \end{array}\right]+\left[\begin{array}{cc} k_{2} & -k_{2}\\ -k_{2} & k_{2}+k_{1} \end{array}\right]\left[\begin{array}{c} z_{2}\\ z_{1} \end{array}\right]=\left[\begin{array}{c} 0\\ \begin{array}{c} k_{1}z_{0}+c_{1}\dot{z_{0}} \end{array} \end{array}\right],$$

where *m*_1_ and *m*_2_ represent the unsprung and sprung mass, respectively; *k*_1_ and *k*_2_ denote the stiffness of the suspension and tire, respectively; *c*_1_ and *c*_2_ correspond to the damping coefficients for the suspension and tire, respectively; *z*_0_, *z*_1,_ and *z*_2_ indicate the vertical displacements of the ground, the unsprung system, and the sprung system, respectively.

The vehicle–road interaction model serves as the foundation for the subsequent experimental design. It allows for the simulation of dynamic loads under various conditions, such as different vehicle speeds and road surface roughness grades. Taking a one-quarter truck as an example (vehicle parameters are shown as Table 1), the truck is fully loaded and traversing a 5 m long road with a surface roughness grade of Class B with speeds of 20 km/h, 40 km/h, 60 km/h, and 80 km/h. Taking the Dynamic Load Coefficient (DLC) as an index (Equation (2)), the DLC is, respectively, 33.5%, 46.7%, 56.2%, and 64.8%, highlighting the influence of vehicle speed on pavement-induced vibrations (as shown in Figure 1b).

(2) $$DLC=\frac{1}{F_{s}}\sqrt{\frac{\sum_{i=1}^{N}{{(F}_{d\_i}-F_{s})}^{2}}{N-1}},\;$$

where N is the number of the samples, $F_{s}$ is the static load, $F_{d\_i}$ is the dynamic load at sample _i.

By establishing a simplified yet effective vehicle–road interaction framework, this section lays the foundation for further investigations into pavement vibration responses and indirect vehicle load estimation based on measured road surface accelerations.

#### 2.1.2. Pavement Vibration Distribution in Lateral and Vertical Direction

The distribution of pavement vibration within layered structures critically influences the optimal placement of vibration sensors and the inverse analysis of load identification. Xue et al. [32] analyzed transverse attenuation of longitudinal strain following a Gaussian function. Yan et al. [33] proposed that quartic polynomial fitting outperformed exponential functions in describing lateral attenuation (maximum R^2^ = 0.969), though its complexity hindered practical application due to excessive parameters.

**Figure 2:**
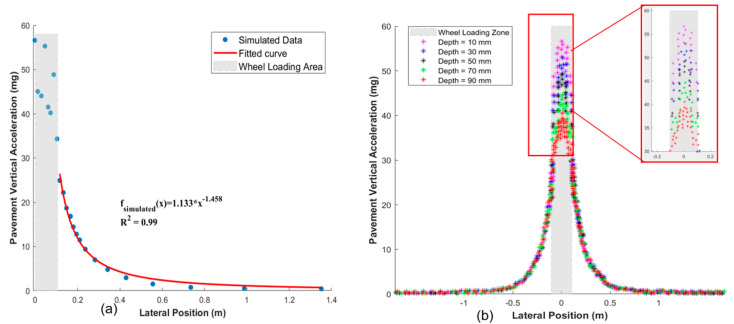
Pavement acceleration distribution by FEM simulation: (**a**) Pavement vertical acceleration distribution in lateral direction (depth = 10 mm). (**b**) Pavement vertical acceleration distribution in different depths (Note: the original simulated data is from the literature [33]).

To overcome this limitation, this study proposes a simplified power-law model for roadside vibration monitoring:

*A*(*x*) = *A*_0_·*x*^−*k*^, (3)

where *A*(*x*) denotes vibration amplitude at lateral distance *x*, *A*_0_ the reference amplitude, and *k* the attenuation exponent. By excluding data within the direct wheel-loading zone, this model achieved consistent accuracy (R^2^ > 0.99) across all pavement layers (as shown in Figure 2). The FE models in the literature are summarized in Table 2, with parameters and validation details.

### 2.2. Full-Scale Accelerated Pavement Testing

#### 2.2.1. Field Test of Full-Scale Accelerated Pavement Testing

**Figure 3:**
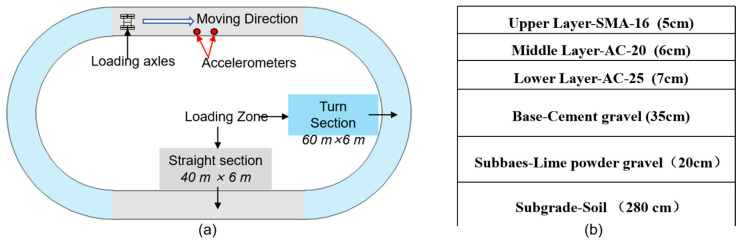
(**a**) Layout of the full-scale Accelerated Loading Test. (**b**) Pavement structure and materials.

**Figure 4:**
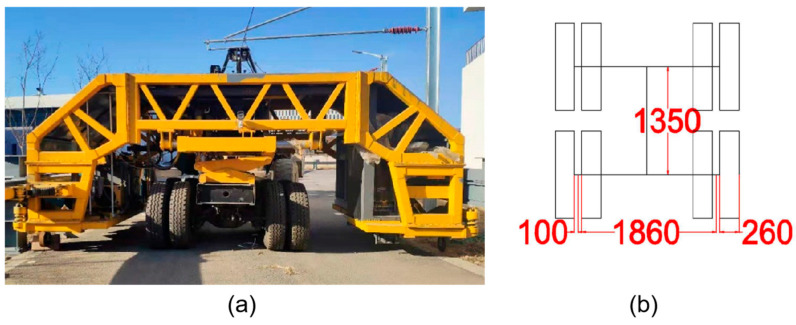
(**a**) Front view of the Loading axles. (**b**) Top view and the size of the frame (unit: mm).

To validate the attenuation pattern of pavement vibration derived from the literature and assess the feasibility of lane-side accelerometers for vehicle load detection, the field test was conducted using the full-scale Accelerated Pavement Testing system. The system is located at the National Center for Materials Service Safety (NCMS), University of Science and Technology Beijing, China. The layout of the system is shown as Figure 3a, and the pavement structure and materials are shown in Figure 3b. The test track has a width of 6 m and a total length of 200 m, which includes two straight sections of 40 m and two curved sections of 60 m. There are two kinds of loading axle: the single axle and the tandem axles. The tandem axles are adopted in this research, taken as the vehicle simulator, which is one of the most widely used standard axle type for trucks in China. The photo and the frame are illustrated as Figure 4. This system can provide loads with a range of 10,000–28,000 kg, loading speeds of 0–30 km/h, and lateral displacements of 0–500 mm. Experiments are conducted under three sets of conditions: varying speeds, different lateral positions, and different axle loads, as shown in Table 3.

**Figure 5:**
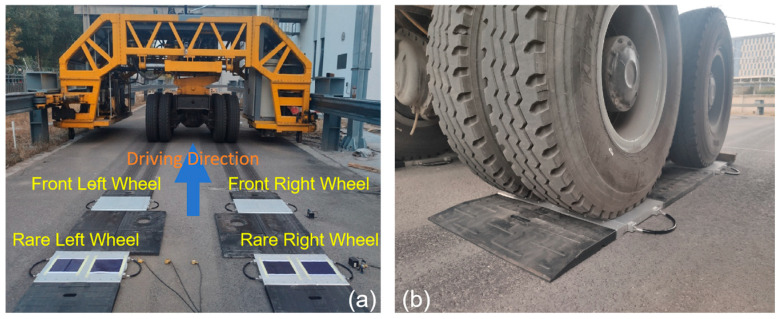
Measuring the static loads of the hydraulic loading system: (**a**) the static load weighing instrument; (**b**) the left wheels in the weighing process.

**Figure 6:**
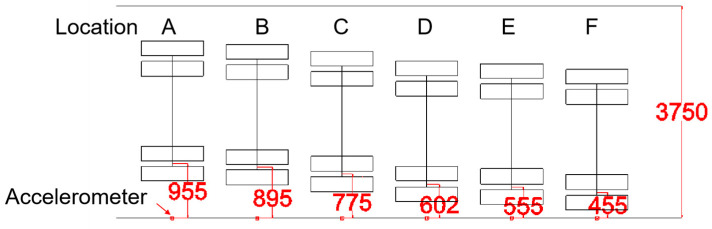
Lateral positions in the field tests (unit: mm).

To determine the static wheel loads under various loading conditions of the hydraulic loading system, a quasi-static axle weighing instrument (type name: HLDB) is employed, as shown in Figure 5. This instrument utilizes a high-strength aluminum alloy weighing table equipped with multiple sensors to weigh each wheel individually, widely used by traffic police and road administration departments for rapid detection of overloaded vehicles. The relationship between hydraulic loading pressure and the static loads of each wheel and the entire vehicle is presented in Table 4, which allows for linear interpolation. Additionally, vehicle speeds are measured using strain sensors embedded in the pavement beneath the wheel path [34] (Table 5), while lateral positions are detailed in Table 6 and Figure 6. To ensure vehicle driving direction and speed uniform during each test, the sensors are positioned at the geometric center of the linear test section within the APT (Accelerated Pavement Testing) facility. Considering the total width of the loading axles (2480 mm) and lane width in an actual road (around 3750 mm), the lateral distance between the accelerometers and the right loading wheel is set from 955 mm to 455 mm in this study, which can cover most of the situations in practice.

#### 2.2.2. Layout of the MEMS Accelerometer

Since the pavement is subject to vehicle loads, sunshine, rain, and temperature difference for a long time, the sensors laid on or embedded in the pavement structure need to meet the requirements of the pavement structure dynamic responses under different conditions. According to the FE analysis and measured data in the field by several researchers, the frequency of asphalt pavement induced by vehicles is general between 10 and 20 Hz [35,36]. And the accelerometer varies within ±250 mg under the of heavy trucks [37], while the peak vibration measured at the monitoring point is about ±10 mg under the action of small cars or away from the center of the wheel load [38]. In this study, the loading axle distance is 1350 mm, and the loading speed ranges from 4.72 km/h to 22 km/h, which indicates that the loading frequency is not exceeding 10 Hz.

**Figure 7:**
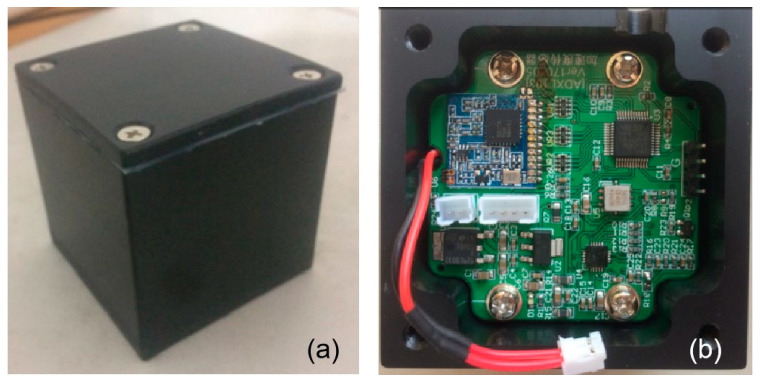
The pictures of the MEMS accelerometer. (**a**) the outlook; (**b**) the inside structure.

Thus, this study selects the MEMS accelerometer that is based on MS9002 developed by our team for the application of array and wireless monitoring. The parameters of the accelerometer are shown in Table 7, and the performance tests have been conducted in the laboratory and field [39]. It is composed of silicon components, a signal processor, a micro-controller and a temperature sensor, of good temperature adaptability and stability through packaging, and its structure and outlook in Figure 7.

**Figure 8:**
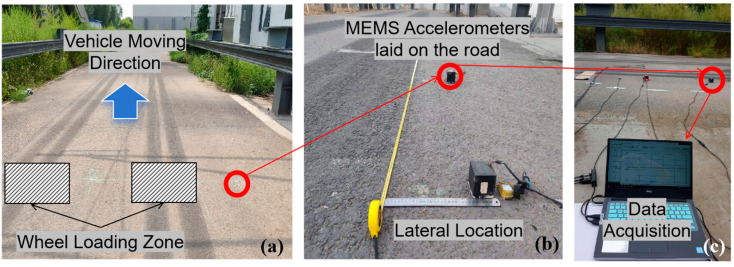
The deployment of the MEMS Accelerometer: (**a**) the deployment of the test field; (**b**) the location of the sensors with measuring the initial lateral distance; (**c**) data acquisition during the test.

The deployment of the sensors is shown in Figure 8. Firstly, mark the lateral positions according to the layout in Figure 3a and Figure 6 (Location A). Then, use the epoxy (DP420 produced by 3M Corp., St. Paul, MN, USA) to fix the two accelerometers to the specified positions at an interval distance of 1 m along the driving direction, to ensure that all vibration signals can be collected. Before fixing, remove loose particles on the pavement surface at the positioning point, and make sure that the monitoring direction of the single-axis accelerometer is vertical, as shown in Figure 8b.

### 2.3. Vibration Signal Acquisition and Processing

The vertical acceleration signals of the pavement are derived from the lane-side MEMS accelerometers, which incorporate a 50 Hz low-pass filter and continuously collect data during the acquisition process, with a sampling rate set at 1000 Hz. Given the characteristics of the signals, to facilitate the extraction of feature values and analysis of patterns, this paper sequentially applies several processing techniques to the raw acceleration signals, to minimize the impact of interference noise. The steps include the following:

(1) Data windowing.

Based on the spatial dimensions of the loading zone and average loading velocity, a 10 s time window (5 s pre-trigger and 5 s post-trigger) centered on the signal occurrence was established for analysis. A rectangular window function was applied to extract raw signal segments.

(2) Baseline correction.

Baseline correction was performed by subtracting the mean value from the signal, effectively removing any DC offset and aligning the signal with zero.

(3) Data filtering.

Smoothing filters were iteratively applied to attenuate trend-related noise while retaining transient signal features.

(4) Feature value extraction.

**Figure 9:**
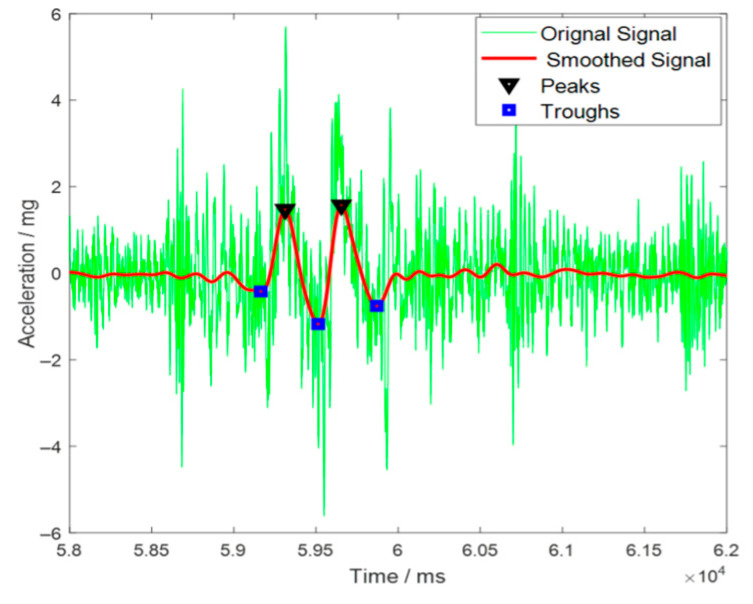
Acceleration Signal Processing.

Peak acceleration was identified as the primary characteristic parameter, defined as the maximum absolute value within each processed window, as shown in Figure 9.

## 3. Experimental Results and Discussion

### 3.1. Acceleration Under Different Loading Speeds

**Figure 10:**
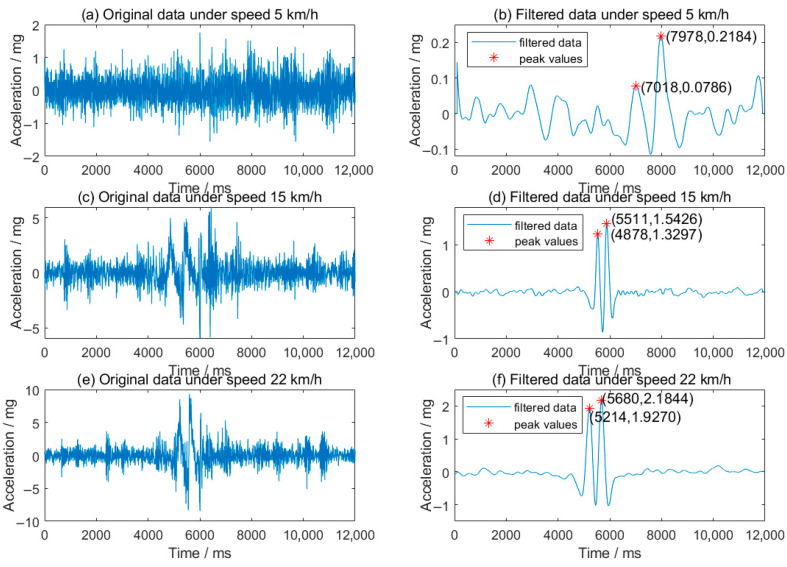
Original and filtered vertical acceleration signals at different speeds.

In this research, the loading speed varies from 5 km/h to 22 km/h; the origin signals of the roadside MEMS accelerometer are shown in Figure 10. When the load speed is low (as shown in Figure 10a), the original vibration signal caused by the vehicle is difficult to differentiate from the noise, primarily due to limited dynamic loading and the distance between the wheel and the sensor. As speed increases, both the original and filtered signals become more discernible.

**Figure 11:**
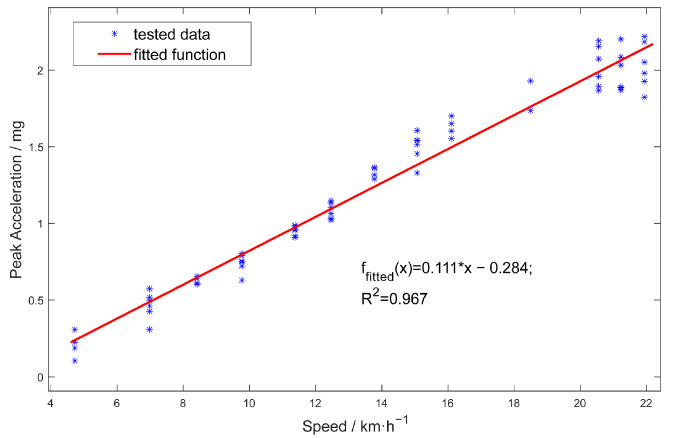
Vertical acceleration trends versus speeds (5–22 km/h).

By extracting the peak accelerations generated by the front and rear axles, the trend of peak acceleration at various speeds is depicted in Figure 11. This figure illustrates the peak vertical acceleration as a function of vehicle speed, spanning from 5 to 22 km/h. The measured data (blue markers) closely align with the fitted linear function (red line), which has a coefficient of determination (R^2^) of 0.967, indicating a strong positive correlation between speed and peak acceleration.

### 3.2. Acceleration Under Loads with Different Lateral Locations (Wandering)

The lateral position of vehicles within a traffic lane is inevitably influenced by multiple factors, serving as a critical indicator of driving safety. Given the limited size of the MEMS sensors, it is essential to quantify the impact of lateral vehicle wander on pavement vibration characteristics.

**Figure 12:**
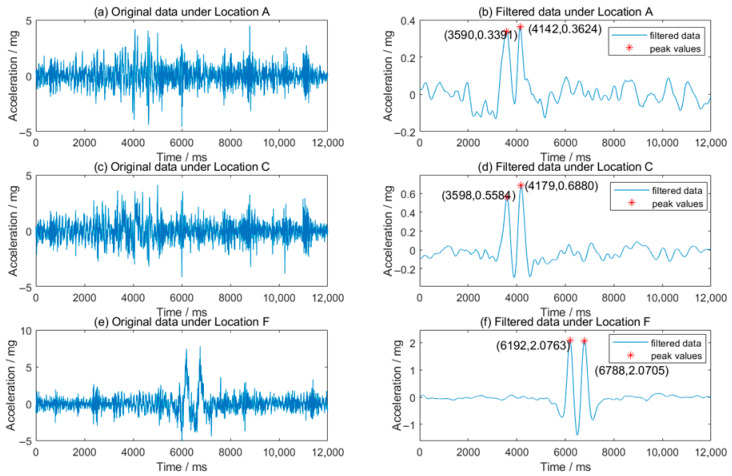
Original and filtered vertical acceleration signals at different lateral positions.

In this study, vehicle loads were applied across six lateral positions (A–F) using an Accelerated Pavement Testing (APT) system, covering a lateral displacement range of 0–500 mm. As described in Section 2.2, Position F represents the closest proximity to the wheel path. Tests were conducted at a constant speed of 16 km/h with a hydraulic pressure of 9 MPa, corresponding to a total vehicle load of 24,666 kg. The original data is shown in Figure 12a,c,e, and the filter data is shown in Figure 12b,d,f. As the loading wheel moves closer to the sensors, both the signal magnitude increases, and the wave shape becomes more regular.

**Figure 13:**
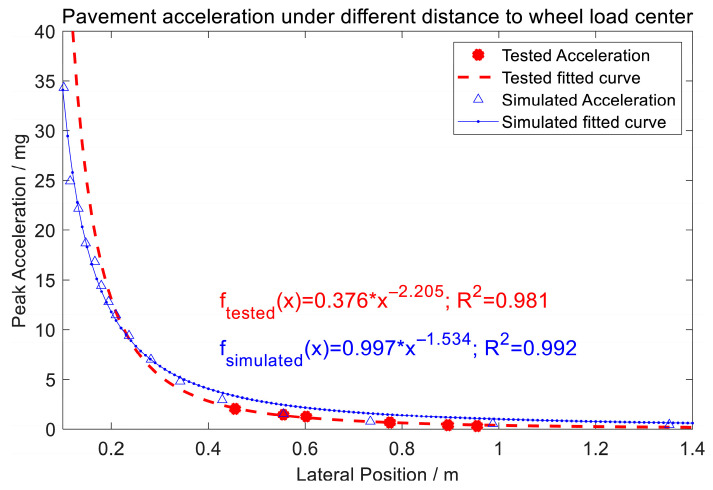
Vertical acceleration trends versus lateral position (0.455–0.955 m).

Figure 13 presents the relationship between pavement acceleration and the lateral distance from the wheel load center. The tested acceleration data (red circles) and its fitted curve (red dashed line) follow a power function and a high coefficient of determination (R^2^ = 0.981). Similarly, the simulated acceleration data (blue triangles) and its fitted curve (blue solid line) exhibit a comparable trend, with R^2^ = 0.992. Both experimental and simulated results demonstrate that peak acceleration sharply decreases with distance, which can be fitted using a power function. These findings provide actionable insights for optimizing MEMS accelerometer deployment in field applications.

### 3.3. Acceleration Under Different Loads

At a speed of 16 km/h, the hydraulic pressure of the loading system varies from 8.5 MPa to 10.5 MPa in increments of 0.2 MPa. The corresponding whole load of the vehicle is from 23,730 to 28,570 kg, while the wheel load ranges from 5400 kg to 7700 kg. The lateral loading position is at Location C, and the distance between the accelerometers and the wheel loading center is 775 mm. Given the relatively high initial load and the limited intervals, a frequency analysis is included in this section to enhance understanding.

**Figure 14:**
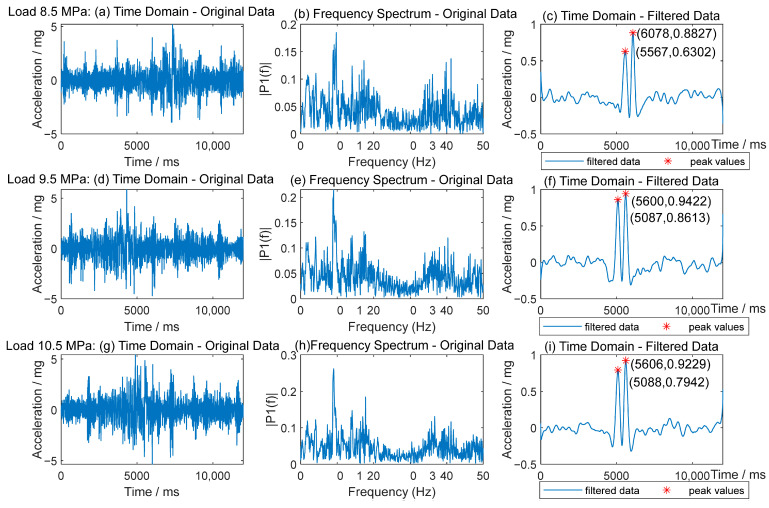
Time-frequency domain signals of vertical acceleration at different vehicle loads.

Figure 14 illustrates the time-frequency characteristics of vertical acceleration under different vehicle loads, ranging from 8.5 MPa to 10.5 MPa. The left column presents the original time-domain acceleration signals, while the middle column shows their corresponding frequency spectra. The right column displays the filtered time-domain data, with peak acceleration values marked for each loading condition. As the hydraulic pressure increases, the magnitude of acceleration signals becomes more pronounced, and the dominant frequency components remain consistent (around 10 Hz). These results highlight the influence of vehicle load on pavement vibration characteristics.

**Figure 15:**
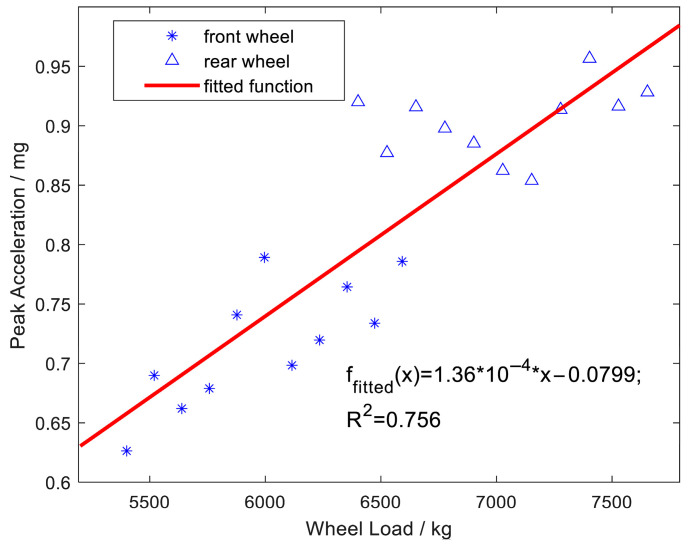
Vertical peak acceleration trends versus wheel load (5400–7700 kg).

Figure 15 presents the relationship between vertical peak acceleration and wheel load, ranging from 5400 kg to 7700 kg. The blue markers represent the measured data for the front and rear wheels, while the red line denotes the fitted function. The fitted equation exhibits a coefficient of determination (R^2^) of 0.756, indicating a moderate correlation. The trend suggests that as the wheel load increases, peak acceleration also rises, with variations between the front and rear wheels. This result highlights the influence of wheel load on pavement acceleration response.

As can be seen from Figure 14a,d,g, neither the original signal nor the peak acceleration is significantly different from the other while the wheel loads are high, and the gap is small. While filtering will highlight the double-peak information to facilitate calculation of axle and speed, it will also lead to signal loss. Therefore, the relationship between vibration signal and vehicle load is analyzed from the perspective of vibration energy. Vibration energy refers to the squared amplitude of the signal, which is always used as an index to analyze the characteristic of vibration signals [37,40]. The energy E in the time domain can be estimated from the sum of squares of the signal sample.

(4) $$E=\sum_{n=0}^{N-1}{\left|x[n]\right|}^{2},$$

where *x*[*n*] is the sample *n* of the signal and *N* is the total number of samples.

In the frequency domain, the vibration energy can also be obtained by calculating the sum of squares over the entire spectrum, which according to Parseval’s theorem is equivalent to the total energy in the time domain:

(5) $$E=\frac{1}{N}\sum_{k=0}^{N-1}{\left|X[k]\right|}^{2},$$

where *X*[*k*] is the FFT result of the signal *k*, and *N* is the total number of samples.

**Figure 16:**
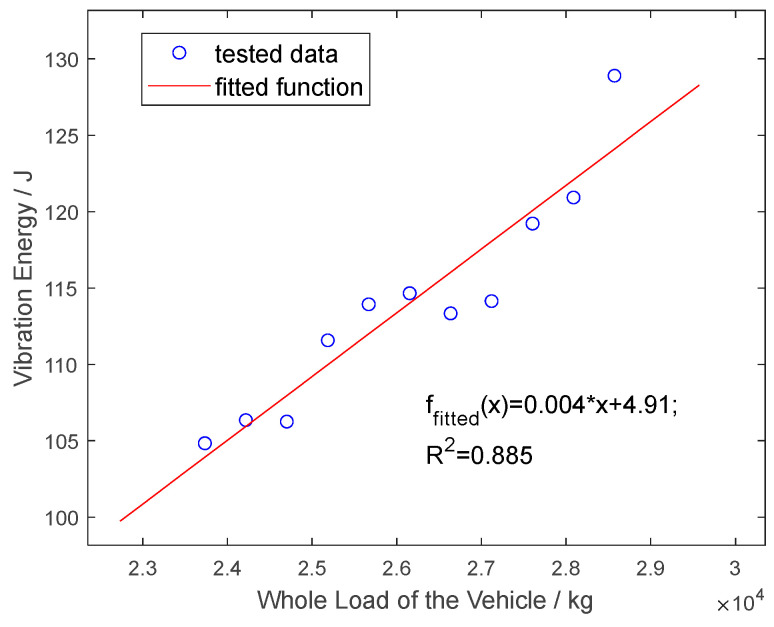
Vertical vibration energy trends versus whole load of the vehicle (23,730–28,570 kg).

Based on the formula above, the vibration energy under different vehicle operating conditions is calculated and linearly fitted to the vehicle load, as shown in Figure 16.

As can be seen from Figure 16, the pavement vibration energy is linearly correlated with vehicle load under a constant vehicle speed, and the correlation can reach 0.885. That is to say, the vehicle load can be derived from the pavement roadside vibration energy.

## 4. Conclusions

This study investigates the feasibility of using roadside MEMS vibration sensors to monitor vehicle load, leveraging a vehicle–pavement coupled vibration model and analyzing the distribution of pavement vibrations under dynamic vehicle loads. By utilizing full-scale accelerated loading equipment and MEMS sensors, vibration data was successfully collected across varying vehicle speeds, lateral displacements, and load conditions. The key findings include the following:

(1) The peak acceleration of roadside vibrations exhibits a strong linear correlation with vehicle speed in the range of 5–22 km/h, with a high correlation coefficient (R^2^ = 96.5%).
(2) As the lateral distance between the wheel center and the monitoring point increases, the peak acceleration decreases following a power law, with a fitting coefficient of 98%, which is consistent with the FEM simulation results.
(3) The relationship between the peak acceleration and the wheel load is approximately linear, with a fitting coefficient of 75.6%. Taking the vibration energy of the original signal as an index, there is a positive linear correlation between the vibration energy and the total vehicle load of the tandem axles, with a correlation coefficient of 88.5%, validating the feasibility of monitoring vehicle loads using roadside vibration sensors.

In conclusion, this study demonstrates the feasibility of roadside MEMS vibration sensors for vehicle load monitoring, providing a cost-effective solution to estimate axle loads, optimize sensor placement, and enhance pavement health assessment. By correlating vibration signatures with vehicle parameters (speed, load), the proposed method advances intelligent transportation systems through scalable infrastructure-integrated sensing.

## Figures and Tables

**Table 1:** Parameters of a truck model. [31].

Unsprung Mass *m*_1_/kg	Sprung Mass *m*_2_/kg	Unsprung Damping *c*_1_/(kN·s·m^−1^)	Sprung Damping *c_2_*/(kN·s·m^−1^)	Unsprung Stiffness *k*_1_/(MN·m^−1^)	Sprung Stiffness *k*_2_/(MN·m^−1^)
550	4450	2	15	1.75	1

**Table 2:** The FE models in the literature.

Authors	Index	Model Size (L·W·H) mm	Element	Materials	Load	Validation
Xue et al. [32]	Horizontal strain	2540 × 1828.8 × 254	C3D8	The top layer is viscoelastic, using the dynamic master curve from laboratory tests, while sublayers are elastic.	Elliptical uniform	Validated by the embedded horizontal strain in the field.
Yan et al. [33]	Vertical acceleration	7500 × 3750 × 3750	C3D8R	Elastic with Rayleigh damping model.	Rectangular nonuniformly distributed	With a speed of 10 m/s, a total load of 2000 kg and a monitoring depth of 70 mm, the simulated and measured peak acceleration are both 45 mg and the time-history curves are similar.

Note that: 1 g is taken as 9.8 m/s^2^, and 1 mg = 9.8 × 10^−3^ m/s^2^.

**Table 3:** Set up of the field tests.

Group No.	Revolutions per Minute	Hydraulic Loading Pressure (MPa)	Lateral Loading Position (mm)
1	140/200/240/280/330/360/400/440/470/540/600/620/640	9	180
2	540	8.5/8.7/8.9/9.1/9.3/9.5/9.7/9.9/10.1/10.3/10.5	180
3	470	9	0/60/180/353/400/500

**Table 4:** Static loads corresponding to different hydraulic loading pressure.

No.	Hydraulic Loading Pressure (MPa)	Whole Load of the Tandem Axles (kg)	Front Left Wheel (kg)	Front Right Wheel (kg)	Rare Left Wheel (kg)	Rare Right Wheel (kg)
1	8	22,643.3	5163.3	5576.7	6093.3	5810
2	8.5	24,043.4	5470	5936.7	6446.7	6190
3	9	24,663.3	5600	5996.7	6683.3	6383.3
4	9.5	25,840	5913.3	6370	6960	6596.7
5	10	27,563.4	6376.7	6763.3	7396.7	7026.7
6	10.5	28,497.5	6572.5	7010	7665	7250
7	11	29,745	6820	7310	7960	7655

**Table 5:** Measured speeds corresponding to different motor speeds by the strain sensors [34] embedded in the pavement beneath the wheel path.

No.	Revolutions per Minute (r/min)	Measured Speed (km/h)	No.	Revolutions per Minute (r/min)	Measured Speed (km/h)
1	140	4.72	8	440	15.07
2	200	6.98	9	470	16.10
3	240	8.42	10	540	18.50
4	280	9.77	11	600	20.55
5	330	11.38	12	620	21.22
6	360	12.47	13	640	21.94
7	400	13.78			

**Table 6:** Lateral position set by the loading system.

Location	Lateral Displacement Set by the Loading System (mm)	Lateral Distance Between the Center of Wheel Loading Zone and the Accelerometer (mm)
A	0	955
B	60	895
C	180	775
D	353	602
E	400	555
F	500	455

**Table 7:** Parameters of the MEMS accelerometer.

Parameter	Description	Parameter	Description
Sensitivity (mV/g)	996.1	Analog Output Bandwidth (Hz)	50
Noise (mV)	0.198	Size (L × W × H, mm)	60 × 60 × 60
Resolution * (mg)	0.199	Maximum Allowable Compressive Strength (MPa)	67.54
Measurement Range (g)	±2	Waterproof Standard Grade	IPX8
Default Sampling Frequency (Hz)	1000		

* Note: the Resolution (0.199 mg) represents the inherent hardware limitation of the MEMS accelerometer. It quantifies the smallest change in acceleration the sensor’s raw output can reliably distinguish before any significant processing. The observed variations can be less than 0.1 mg due to the processed data stream, not a violation of the sensor’s physical limits.

## Data Availability

The raw data supporting the conclusions of this article will be made available by the authors on request.
